# Supplementary material for: TSEN54 missense variant in Standard Schnauzers with leukodystrophy
Source: PLoS Genet. 2019 Oct 4;15(10):e1008411. doi: 10.1371/journal.pgen.1008411 (PMC6795476; doi:10.1371/journal.pgen.1008411)
Supplement: Attachment — Submitted filename: MS_Stoerk__R1_Response_to_Reviewers__v02.pdf [file pgen.1008411.s008.pdf]

Reviewer 1

Comment 1:

There are no gross or even sub gross images to allow readers to evaluate regions variance of the disease. Even just one of these images could help the reader better frame the location and extent of affected tissue. While there is some indication of overall lesions in the MRI images, it does not replace the context that would be provided by whole brain or sectioned brain images.

> **Author's response:** The authors agree with the reviewer, but they regret, that at the time of necropsy, no gross images were taken and formalin-fixed tissue isn't available anymore. Due to the size of the tissue blocks, subgross images of histological slides would not allow to give an overview over the distribution of the lesions. However, following the suggestion of the reviewer to provide a convincing image about the localization of the lesions, the authors provide macroscopic images of one age matched control animal to demonstrate the localization of the lesions.
>
> New image was included as figure 2. Other images had been subsequently renumbered.
>
> „**Fig 2. Macroscopic overview.** (A, B) Age matched healthy control no. 1009. (A) Dorsal view on cerebrum, cerebellum and brain stem with section plane (white line). (B) Cross section of cerebrum showing the *centrum semiovale* (red circle).“ (page 9)

Comment 2:

Differentiating between hypo-myelination verse demyelination is important considering that the dogs had such an early onset of disease. Additional diagnostics such as TEM to evaluate myelin formation abnormalities verses demyelination would be extremely helpful and could be done on formalin fixed tissue, though I admit the fixation and thus image quality would be diminished compared to standard EM fixation protocols.

> **Author's response:** The authors agree with the reviewer, that TEM would be interesting to further investigate the disease and the lesions. However, due to the poor preservation status of tissue (immersion fixation, not perfused tissue) and loss of tissue consistency, tissue preparation resulted in no convincing read outs.

Comment 3:

While CNPase does stain oligodendrocytes, it is a cytoplasmic and nuclear stain that only allows for at best a subjective assessment of the number of oligodendrocytes. In the images provided, the stain quality of the CNPase does not appear to allow for effective quantification of oligodendrocytes. Changes in oligodendrocyte density is an important feature of this form of leukodystrophy, which was clearly described and highlighted in the discussion by the authors. A nuclear stain such as Olig-2 would provide a more clear representation of oligodendrocyte numbers and would complement the CNPase or LFB stain. Subsequent quantitative analysis of a nuclear based stain would also be more helpful in demonstrating the differences in oligodendrocyte density. Given that the authors suggest that this variant of TSEN54 results in dysfunction and early cell death of oligodendrocytes clear quantification is important.

> **Author's response:** The authors agree with the reviewer, that other oligodendrocyte specific stains would allow more precise quantification of the cell type of interest. Currently, the

authors are not using Olig-2 routinely, therefore they decided to use an other oligodendrocyte marker, neurite outgrowth inhibitor A (NogoA). Subsequently NogoA was used to quantify oligodendrocytes. According to literature this is a reliable oligodendroglial marker, too (e. g. Kuhlmann et al. 2007). NogoA immunohistochemistry was evaluated using a morphometric grid and statistical analysis was performed.

Within the manuscript following text passages had been added:

> „We quantified mature myelinating oligodendrocytes using an antibody against neurite outgrowth inhibitor A (NogoA). Within the centrum semiovale, affected Schnauzer puppies show only single NogoA+ oligodendrocytes, whereas age matched healthy control animals show up to eight times as many myelinating NogoA+ oligodendrocytes as the affected animals in this area (Fig 6). " (page 11)

> „**Fig 6. Immunohistochemistry of the white matter in the centrum semiovale of the cerebrum**. (A) Affected puppy no. 1 and (B) age matched healthy control no. 1009. (A) In affected puppies, only single NogoA+ oligodendrocytes are detectable (arrows). Vacuolization and loosening of the parenchyma indicate the moderate edema. Inset shows a NogoA+ oligodendrocyte at a higher magnification. (NogoA). (B) Age matched control animals show numerous NogoA+ myelinating oligodendrocytes. Inset shows NogoA+ oligodendrocytes at a higher magnification. (NogoA). (C) Statistical analysis of NogoA-immunohistochemstry shows significantly decreased numbers of myelinating oligodendrocytes in the centrum semiovale of the affected Schnauzer puppies (n=4, no.1, 2, 3 and 4) in comparison to age matched control animals (n=4, no. 1009, 1010, 1011 and 1012). Box and whisker plots display median and quartiles with maximum and minimum values. Significant difference ($p \leq 0.05$, Mann-Whitney U-test) is labeled by asterisk." (page12)

> „Immunohistochemistry was performed on formalin-fixed, paraffin-embedded (FFPE) sections of cerebrum of affected animals (no. 1-12) and age-matched controls (no. 1009-1016) using monoclonal antibodies directed against 2′,3′-cyclic nucleotide-3′-phosphodiesterase (CNPase; MAB 326, dilution 1:100; Chemicon International), β-amyloid precursor protein (β-APP; MAB 348, dilution 1:800, Chemicon International), and polyclonal antibodies against myelin basic protein (MBP; AB 980, dilution 1:800, Chemicon International), neurite outgrowth inhibitor A (NogoA; AB5664, dilution 1:500, Merck Milipore), glial fibrillary acidic protein (GFAP; Z 0334, dilution 1:200, DAKO) and ionized calcium binding adaptor molecule 1 (Iba-1; PA5-27436, dilution 1:500, Thermo Fisher Scientific Inc.) to detect oligodendrocytes, axonal damage, astrocytes, and macrophages/microglia, respectively."(page 24)

> „For quantitative analysis of mature oligodendrocytes characterized by NogoA-immunohistochemistry, a morphometric grid (number of positive cell/0,0625mm2) was used." (page 25)

„**Statistical analysis**

Statistical analysis of non-normal distributed data generated by NogoA-immunohistochemistry was performed by using IBM "Statistic Package for Social Sciences" SPSS program for Windows (version 24) and applying a Mann-Withney U-test for two independent samples. A p-value of less than or equal to 0.05 was considered to show statistically significant difference between affected and control animals." (page 25)

Comment 4:

The CNPase, MBP and LFB seem redundant in their purpose as they are currently described in this study. From what I can determine they are all being used to evaluate the extent of myelination and demonstrate changes in myelin or myelin associated oligodendrocyte process, which are intimately related. If each of these stains are outlining a distinct feature that is particular to this form of leukodystrophy that should be made clearer in the discussion. For example: LFB is a general myelin histochemical stain whereas the CNPase detects a more early form a myelin.

Author's response: the authors agree with the reviewer, that the different myelin stains may appear redundant. However, as indicated by the reviewer, they allow to detect different stages of myelin formation. To allow the reader to follow the reasoning for using these different myelin stains, the following text passages has been included:

„In addition to the more general histochemical stain for myelin, LFB, two other different myelin stains were applied, using 2',3'-cyclic nucleotide-3'-phosphodiesterase (CNPase) as a marker for early myelin formation and myelin basic protein (MBP) as the prototype of mature myelin formation." (page 10)

„The results of the different myelin stains, underline the leukodystrophic character of the disease of the present cases. CNPase immunohistochemistry detecting both immature oligodendrocytes and early myelin formation showed the presence of oligodendrocytes and the predominant absence of myelin in the *centrum semiovale* of the affected animals. In addition the MBP immunohistochemistry revealed single fine strands of mature myelin in affected areas. This could be interpreted as an insufficient and inadequate myelin formation mediated by the detected gene defect." (page 19)

Comment 5:

APP is not as widely used in veterinary medicine as it is in human medicine for the detection of axon damage and its effect in this disease is not clear in the images provided. In the paper cited by the authors (reference 32) the authors used a counter stain with either LFB-CV or Bielschowsky's silver stain to highlight the APP stain in the context of the myelin or the axons. This could greatly improve the images and show where in the cells and at what areas the axon damage is occurring. In addition, the authors could expand on the importance of the APP findings in the discussion.

Author's response: The authors thank the reviewer for this comment. To visualize the colocalization of axonal damage and lack of myelin, an additional combination of APP immunohistochemistry and LFB-CV staining was performed and added to the manuscript. Figures have been rearranged and the additional staining has been included.

„Using an amyloid precursor protein (APP) specific immunohistochemistry, mild, multifocal axonal damage was noticed in areas of severe lack of myelin (Fig 4C, D). In the combined APP immunohistochemistry and LFB staining the colocalization of axonal damage and myelin loss was visualized (Fig 4E, F).“ (page 10)

„**Fig 4. Histochemistry and immunohistochemistry of the white matter in the centrum semiovale of the cerebrum.** (A, C, E) Affected puppy no. 1 and (B, D, F) age matched healthy control no. 1009. (A) Severe, diffuse lacking bluish staining, indicating myelin loss and edema, in the centrum semiovale (asterisk). Only single fine strands of myelin can be detected (circle). (LFB). (B) Regularly developed myelin characterized by prominent bluish staining in the white matter (dark blue). (LFB). (C) Areas of severe myelin loss in the diseased puppies reveal low numbers of damaged axons (arrows). Vacuolization and loosening of the parenchyma indicate moderate edema. Inset shows a damaged axon at a higher magnification. (APP). (D) No axonal damage is detectable in healthy control animals. (APP). (E) Severe, diffuse lacking blue-greenish staining, indicating myelin loss and edema, in the *centrum semiovale*, containing low numbers of damaged axons (arrows). Vacuolization and loosening of the parenchyma represent the moderate edema. Inset shows a damaged axon at a higher magnification. (APP LFB). (F) Regularly developed myelin characterized by prominent blue-greenish staining in the white matter (dark teal) without any damaged axons. (APP LFB).“ (page 10)

Furthermore, findings in the APP immunohistochemstry were discussed in more detail. Following text passages had been added:

„ However, axonal damage in areas of myelin lack as shown in the present cases, has been reported as an epiphenomenon in leukodystrophies [17]. In several leukodystrophic diseases axonal damage is not directly caused by the lack of myelin or oligodendrocytes and rather represents a secondary event [18]. Several possible mechanisms of axonal damage in white matter diseases, such as sulfatide storage in metachromatic leukodystrophy in humans or aberrant glutamate homeostasis and nitric oxide production in multiple sclerosis have been described [19]. In addition, an impairment of axons as a consequence of demyelination and the asscociated loss of myelin-derived trophic support and ion imbalance according to the outside-in model cannot be excluded [20]. The pathomechanism of axonal damage in *TSEN54* associated leukodystrophy is still unclear and warrants additional investigations. “ (page 18)

„ **Combination of immunohistochemistry and histochemistry**

A combination of immunohistochemistry and histochemistry, using APP and LFB, was performed as described to point out the localization of damaged axons [38]." (page 25)

Comment 6:

I really appreciate the immunofluorescent images in Figure 7 and the authors do a nice job of speculating as to why TSEN54 may cause a leukodystrophy in dogs. It would be interesting to evaluate the effects of this variant and this proteins function in oligodendrocyte differentiation and development in dogs, but this may be beyond the scope of this particular study.

Author's response: The authors agree with this interesting point raised by the reviewer It would be really a scientific challenge and an interesting task to further investigate and elucidate the role of this missense variant of TSEN54 and its function in the development of the CNS in general and especially in dogs. However potential investigations to tackle this complex question require additional experiments, which are unfortunately beyond the scope of this study.

Comment 7:

Be sure and review the all figure legends so they are consistent with the article text. In figure 2, the legend seems to imply that the changes or paleness were only associated with a loss of myelin rather than the likely presence of concurrent edema, which was described in the article.

Author's response: The figure legends were adapted according to the comment of the reviewer.

Fig. 4A "Severe, diffuse lacking bluish staining, indicating myelin loss and edema in the centrum semiovale (asterisk)."

Reviewer 2 did not have any specific requests for changes to the manuscript.
