# Supplementary material for: TSEN54 missense variant in Standard Schnauzers with leukodystrophy
Source: PLoS Genet. 2019 Oct 4;15(10):e1008411. doi: 10.1371/journal.pgen.1008411 (PMC6795476; doi:10.1371/journal.pgen.1008411)
Supplement: Attachment — Submitted filename: ECG comments 2019.07.22.docx [file pgen.1008411.s007.docx]

Reviewer comments: TSEN54 missense variant in Standard Schnauzers with leukodystrophy.

In this article the authors do a nice job of describing their approach to the identification of a missense variant in TSEN54 that results in leukodystrophy in a family of Standard Schnauzers. The approach to the identification of the variant is clear and well described.

Overall the authors did a good job describing the clinical signs and lesions associated with the disease, but I have a few concerns with the pathology of the lesions described.

1) There are no gross or even sub gross images to allow readers to evaluate regions variance of the disease. Even just one of these images could help the reader better frame the location and extent of affected tissue. While there is some indication of overall lesions in the MRI images, it does not replace the context that would be provided by whole brain or sectioned brain images.
2) Differentiating between hypo-myelination verse demyelination is important considering that the dogs had such an early onset of disease. Additional diagnostics such as TEM to evaluate myelin formation abnormalities verses demyelination would be extremely helpful and could be done on formalin fixed tissue, though I admit the fixation and thus image quality would be diminished compared to standard EM fixation protocols.
3) While CNPase does stain oligodendrocytes, it is a cytoplasmic and nuclear stain that only allows for at best a subjective assessment of the number of oligodendrocytes. In the images provided, the stain quality of the CNPase does not appear to allow for effective quantification of oligodendrocytes. Changes in oligodendrocyte density is an important feature of this form of leukodystrophy, which was clearly described and highlighted in the discussion by the authors. A nuclear stain such as Olig-2 would provide a more clear representation of oligodendrocyte numbers and would complement the CNPase or LFB stain. Subsequent quantitative analysis of a nuclear based stain would also be more helpful in demonstrating the differences in oligodendrocyte density. Given that the authors suggest that this variant of TSEN54 results in dysfunction and early cell death of oligodendrocytes clear quantification is important.
4) The CNPase, MBP and LFB seem redundant in their purpose as they are currently described in this study. From what I can determine they are all being used to evaluate the extent of myelination and demonstrate changes in myelin or myelin associated oligodendrocyte process, which are intimately related. If each of these stains are outlining a distinct feature that is particular to this form of leukodystrophy that should be made clearer in the discussion. For example: LFB is a general myelin histochemical stain whereas the CNPase detects a more early form a myelin.
5) APP is not as widely used in veterinary medicine as it is in human medicine for the detection of axon damage and it effect in this disease is not clear in the images provided. In the paper cited by the authors (reference 32) the authors used a counter stain with either LFB-CV or Bielschowsky’s silver stain to highlight the APP stain in the context of the myelin or the axons. This could greatly improve the images and show where in the cells and at what areas the axon damage is occurring. In addition, the authors could expand on the importance of the APP findings in the discussion.
6) I really appreciate the immunoflourescent images in Figure 7 and the authors do a nice job of speculating as to why TSEN54 may cause a leukodystrophy in dogs. It would be interesting to evaluate the effects of this variant and this proteins function in oligodendrocyte differentiation and development in dogs, but this may be beyond the scope of this particular study.
7) Be sure and review the all figure legends so they are consistent with the article text. In figure 2, the legend seems to imply that the changes or paleness were only associated with a loss of myelin rather than the likely presence of concurrent edema, which was described in the article.
